# Supplementary material for: Quantifying genetic heterogeneity between continental populations for human height and body mass index
Source: Sci Rep. 2021 Mar 4;11:5240. doi: 10.1038/s41598-021-84739-z (PMC7933291; doi:10.1038/s41598-021-84739-z)
Supplement: Supplementary file 1 — Supplementary Information [file 41598_2021_84739_MOESM1_ESM.pdf]

Quantifying genetic heterogeneity between continental populations for human height and body mass index

Jing Guo[1,2], Andrew Bakshi[3,4], Ying Wang[1], Longda Jiang[1], Loic Yengo[1], Michael E Goddard[5,6], Peter M Visscher[1,*], Jian Yang[1,7,8,*]

[1] Institute for Molecular Bioscience, The University of Queensland, Brisbane, Queensland 4072, Australia

[2] Human Genetics, Wellcome Sanger Institute, Hinxton, CB10 1SA, UK

[3] Monash Partners Comprehensive Cancer Consortium, Monash Biomedicine Discovery Institute Cancer Program, Prostate Cancer Research Group, Department of Anatomy and Developmental Biology, Monash University, Clayton, Victoria 3800, Australia

[4] Queensland Brain Institute, The University of Queensland, Brisbane, Queensland 4072, Australia

[5] Faculty of Veterinary and Agricultural Science, University of Melbourne, Parkville, Victoria, Australia

[6] Biosciences Research Division, Department of Economic Development, Jobs, Transport and Resources, Bundoora, Victoria, Australia

[7] School of Life Sciences, Westlake University, Hangzhou, Zhejiang 310024, China

[8] Westlake Laboratory of Life Sciences and Biomedicine, Hangzhou, Zhejiang 310024, China

[*] These authors jointly supervised the work.

Correspondence: Jian Yang (jian.yang@westlake.edu.cn)

Supplementary Figures 1-6

Supplementary Tables 1-5

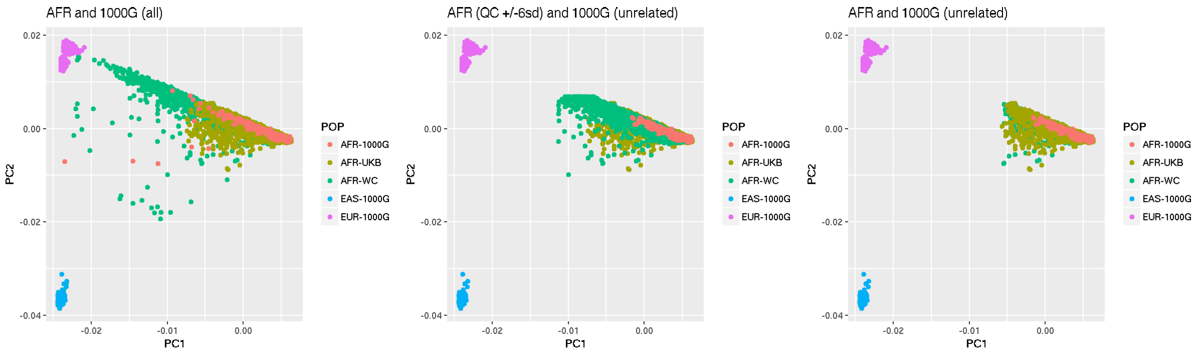

Supplementary Figure 1 Quality control of the AFR (from UKB and WC; Methods) samples using the principal component analysis based on the common HapMap3 SNPs (MAF > 0.01) in the AFR (from UKB and WC) and the 1000 Genomes Project (1000G) combined samples. The left panel shows all the samples in the 1000G and AFR (from UKB and WC) including relatives, the middle one shows the unrelated individuals (estimated pairwise relatedness < 0.05) for each population in the 1000G and the AFR (from UKB and WC) samples removing the individuals who were more than 6 standard deviations (s.d.) away from the mean of the AFR-1000G and the right panel presents the unrelated individuals in the 1000G and AFR (from UKB and WC). The AFR (from UKB and WC) samples in the middle and right panel were used in fastGWA and GREML analyses respectively (Methods). WC indicates WHI and CARe cohorts (Methods).

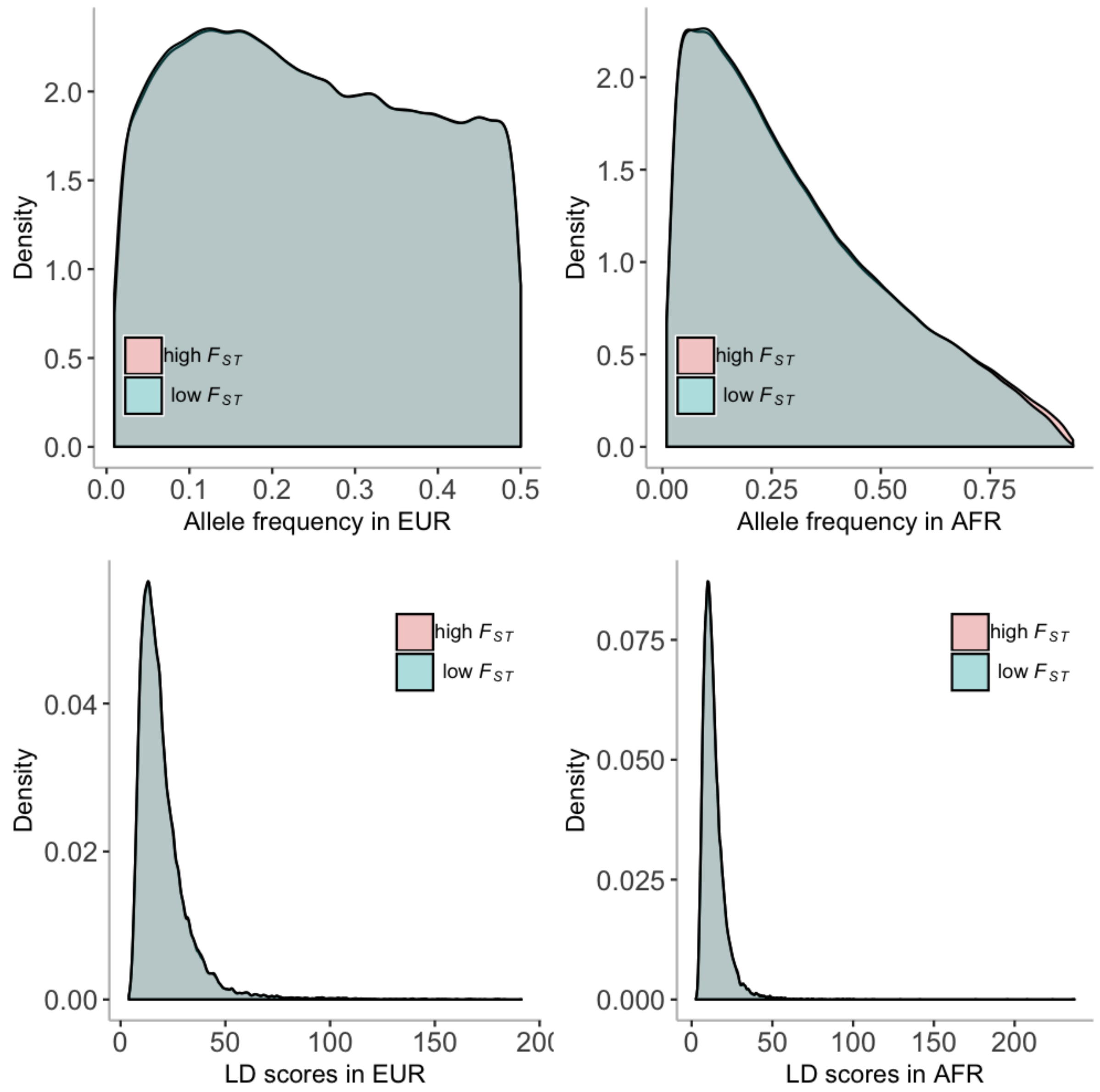

Supplementary Figure 2 Comparison of the distributions of allele frequencies of the minor alleles in EUR and the same alleles in AFR (upper row), and LD scores in EUR and AFR (lower row) between the SNP sets stratified by F_{ST} values. The two distributions in each panel are almost exactly on top of each other. The LD scores shown here are segment-based LD scores by averaging the per-SNP LD scores across SNPs in a 200kb window with 100kb overlap between two adjacent segments (Methods).

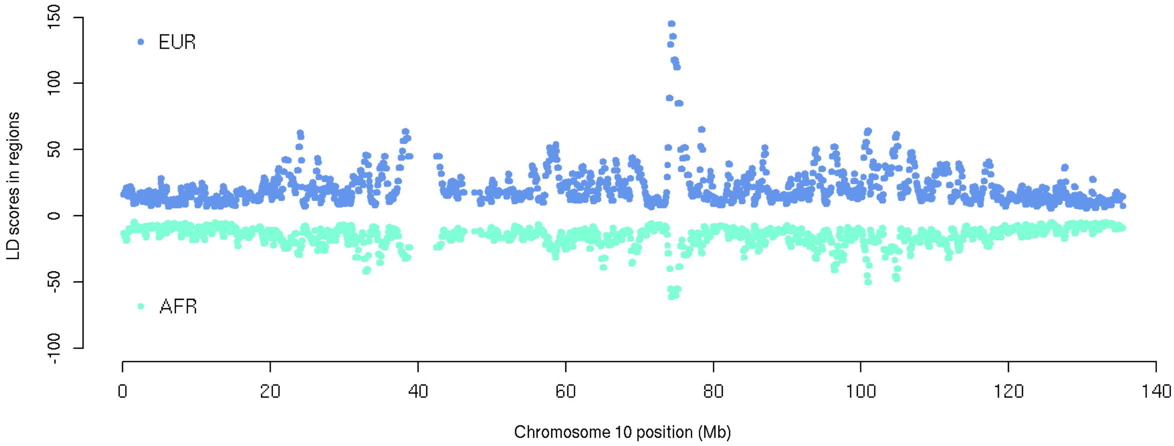

Supplementary Figure 3 An example plot of the LD scores of the HapMap3 SNPs on chromosome 10 estimated in the unrelated EUR and AFR individuals. Each dot indicates the value of a segment-based LD score by averaging the per-SNP LD scores across SNPs in a 200kb window with 100kb overlap between two adjacent segments (Methods).

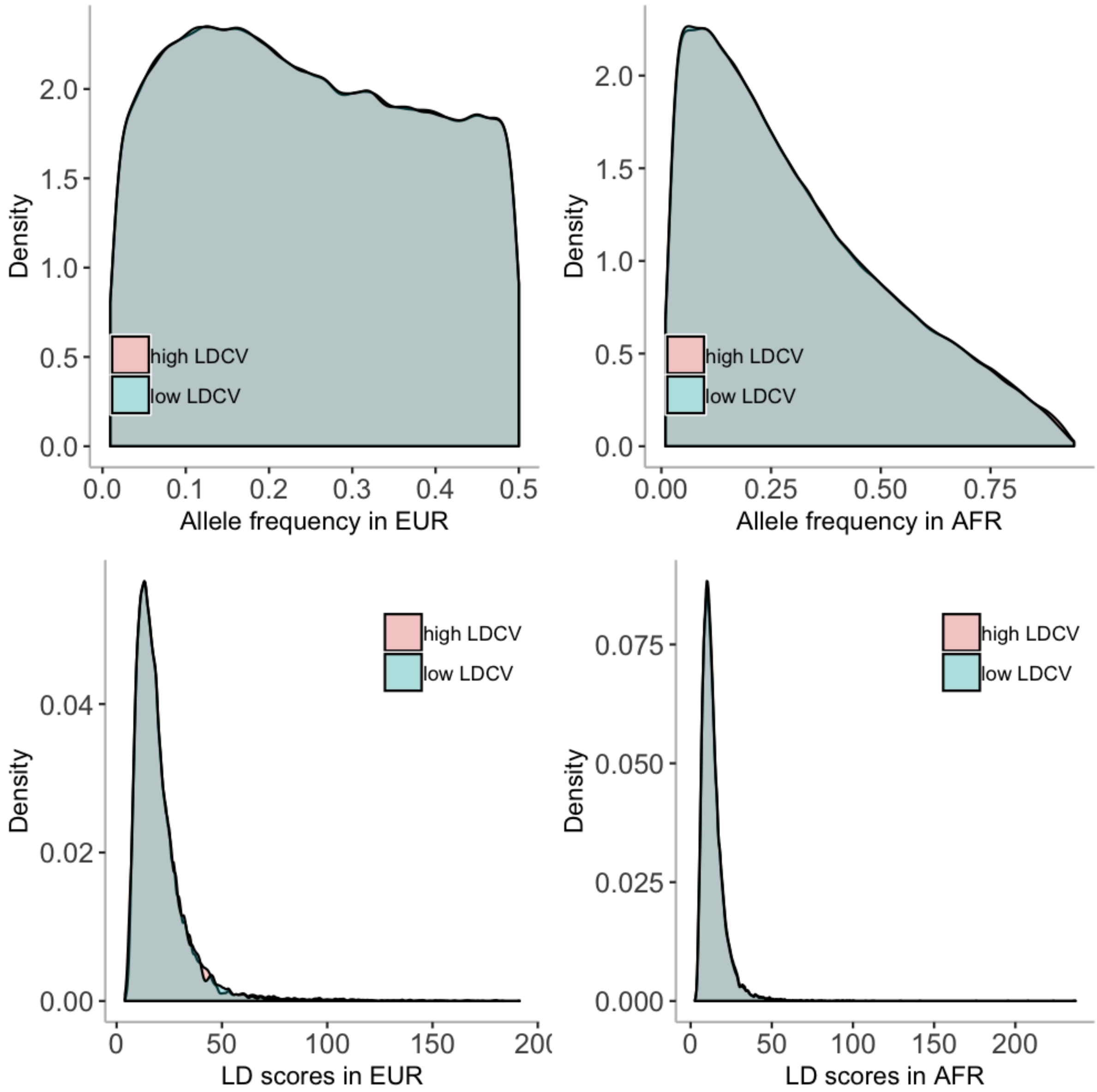

Supplementary Figure 4 Comparison of the distributions of allele frequencies at the minor alleles in EUR and the same alleles in AFR (upper row), and LD scores in EUR and AFR (lower row) between the SNP sets stratified by LDCV values. The two distributions in each panel are almost exactly on top of each other. The LD scores shown here are segment-based LD scores by averaging the per-SNP LD scores across SNPs in a 200kb window with 100kb overlap between two adjacent segments (Methods).

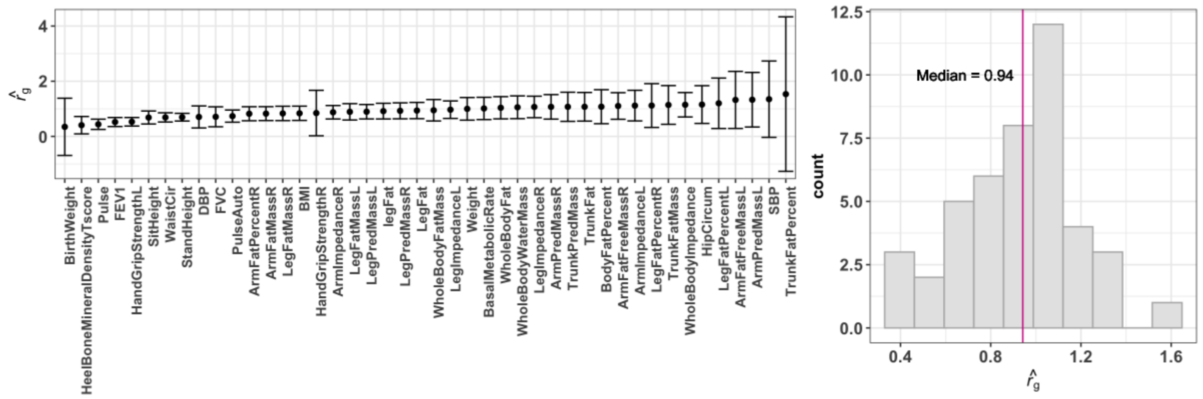

Supplementary Figure 5 The estimated $\hat{r}_g$ (s.e.) between UKB-EUR (n = ~450k) and UKB-AFR (n = ~6,300) for 44 quantitative traits (including height and BMI) using the summary data-based approach (left panel). The distribution of the $\hat{r}_g$ is shown in the right panel.

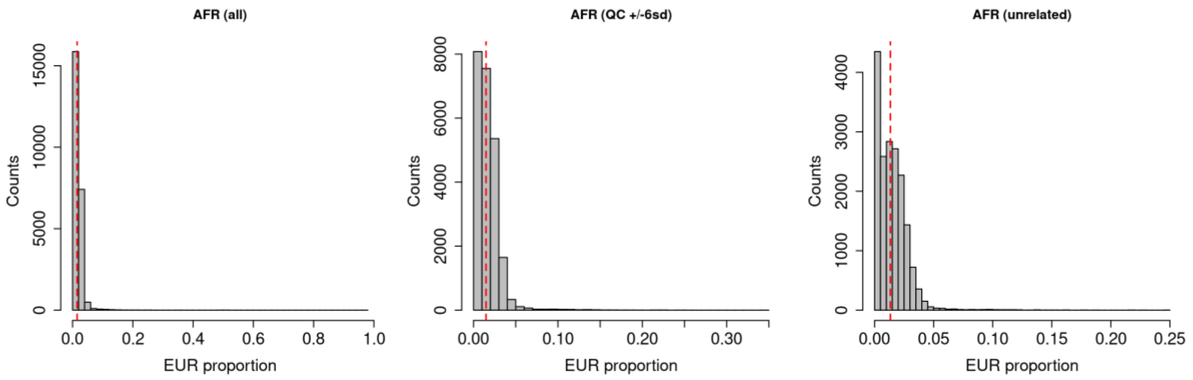

Supplementary Figure 6 Distribution of the estimated percentage of European ancestry using ADMIXTURE in our AFR data before QC, AFR after QC (outlier removal), and the unrelated AFR after QC, corresponding to the 3 panels in **Supplementary Figure 1**. The median values of the three distributions are 0.015, 0.014 and 0.013, respectively (highlighted in red).

Supplementary Table 1 The estimated $\hat{r}_g$ between EUR and AFR using HapMap3 SNPs based on the GRMs-average for height and BMI.

	$\hat{h}^2_{\mathrm{EUR}}$ **(s.e.)**	$\hat{h}^2_{\mathrm{AFR}}$ **(s.e.)**	$\hat{r}_g$ **(s.e.)**
**Height**	0.51 (0.0076)	0.39 (0.024)	0.78 (0.036)
**BMI**	0.26 (0.0081)	0.21 (0.024)	0.72 (0.067)

Supplementary Table 2 The estimated $\hat{h}^2_{SNP}$ and $\hat{r}_g$ between EUR and AFR using HapMap3 SNPs based on the GRMs-specific for height and BMI without fitting PCs as covariates.

	$\hat{h}^2_{\mathrm{EUR}}$ **(s.e.)**	$\hat{h}^2_{\mathrm{AFR}}$ **(s.e.)**	$\hat{r}_g$ **(s.e.)**
**Height**	0.50 (0.0077)	0.39 (0.024)	0.75 (0.035)
**BMI**	0.25 (0.0080)	0.22 (0.024)	0.69 (0.062)

Supplementary Table 3 Estimated $\hat{h}^2_{SNP}$ for height using the HapMap3 SNPs that are on (m=185k) and not on (m=832k) the Affymetrix Human Origin array, respectively.

**SNP set**	$\boldsymbol{h^2_{\mathrm{EUR}}}$ **(SE)**	$\boldsymbol{h^2_{\mathrm{AFR}}}$ **(SE)**	$\boldsymbol{P_{\mathrm{difference}}}$
SNPs on Human Origin array	0.14 (0.012)	0.13 (0.031)	0.77
SNPs not on Human Origin array	0.36 (0.014)	0.26 (0.035)	0.0099

Supplementary Table 4 Estimated $\hat{r}_g$ between AFR and EUR based on the ancestry specific GRMs for height and BMI excluding AFR individuals with the estimated proportions of EUR ancestry lower than 0.1.

	$\hat{h}^2_{\mathrm{EUR}}$ (s.e.)	$\hat{h}^2_{\mathrm{AFR}}$ (s.e.)	$\hat{r}_g$ (s.e.)
**Height**	0.50 (0.0077)	0.39 (0.024)	0.75 (0.035)
**BMI**	0.25 (0.0080)	0.22 (0.025)	0.68 (0.062)

Supplementary Table 5 Estimated $\hat{r}_g$ between EUR and AFR based on the ancestry specific GRMs for height and BMI with the AFR individuals stratified into two groups with lower and higher proportions of European ancestry.

	**EUR proportion**	$\hat{r}_g$ **(s.e.)**
**Height**	lower	0.69 (0.060)
	higher	0.78 (0.058)
**BMI**	lower	0.53 (0.084)
	higher	0.74 (0.12)
